# 5-Sulfanyl­idene-2*H*,5*H*-1,3-dithiolo[4,5-*d*][1,3]dithiol-2-one

**DOI:** 10.1107/S1600536812005703

**Published:** 2012-02-17

**Authors:** Hua-Wen Wen, Li-Xia Fan, Li Liu, Ao Li, Qi Fang

**Affiliations:** a School of Chemistry and Chemical Engineering, Shandong University, Jinan 250100, Shandong Province, People’s Republic of China; b State Key Laboratory of Crystal Materials, Shandong University, Jinan 250100, Shandong Province, People’s Republic of China

## Abstract

The title mol­ecule, C_4_OS_5_, is essentially planar, with an r.m.s. deviation of 0.032 (3) Å. All the C—S single bonds are shorter than the standard C*sp* ^3^—S single-bond length, showing the π-conjugated nature of the molecule. In the crystal, molecules lie parallel to one another and pack in columns along the *a* axis. Short inter­molecular S⋯S contacts [3.314 (3), 3.482 (2) and 3.501 (2) Å] are observed between the columns. The angle between the two mol­ecular dipole moments in the unit cell is 39.3 (1)° and the macro-polarization vector is along the [1 0 − 1.41] direction. As a result of the high polarization and π-conjugation of the structure, the crystalline powder exhibits a second harmonic generating intensity, which is as strong as that of the urea standard powder crystals, when irradiated by a 1053 nm laser beam. The diffraction space of the crystal showed a nonmerohedral twinning.

## Related literature

For details of *GAUSSIAN03* software, see: Frisch *et al.* (2003 ▶). For the synthesis, see: Schumaker & Engler (1977 ▶); Wang *et al.* (1998 ▶).
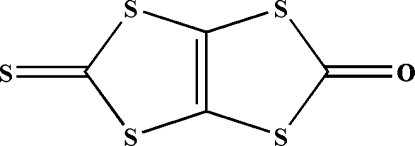


## Experimental

### 

#### Crystal data

C_4_OS_5_
*M* *_r_* = 224.34
Monoclinic, 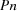

*a* = 3.9638 (2) Å
*b* = 11.0211 (8) Å
*c* = 8.7110 (7) Å
β = 101.344 (5)°
*V* = 373.11 (4) Å^3^
*Z* = 2
Mo *K*α radiation
μ = 1.47 mm^−1^
*T* = 294 K
0.27 × 0.07 × 0.05 mm

#### Data collection

Bruker APEXII CCD diffractometer
Absorption correction: multi-scan (*TWINABS*; Bruker, 2005 ▶) *T*_min_ = 0.697, *T*_max_ = 0.928
8524 measured reflections
1683 independent reflections
1487 reflections with *I* > 2σ(*I*)
*R*_int_ = 0.039

#### Refinement

*R*[*F* ^2^ > 2σ(*F* ^2^)] = 0.044
*wR*(*F* ^2^) = 0.130
*S* = 1.12
1683 reflections
92 parameters
2 restraints
Δρ_max_ = 0.41 e Å^−3^
Δρ_min_ = −0.45 e Å^−3^
Absolute structure: Flack (1983 ▶), 815 Friedel pairs
Flack parameter: 0.1 (3)

### 

Data collection: *APEX2* (Bruker, 2005 ▶); cell refinement: *SAINT* (Bruker, 2005 ▶); data reduction: *SAINT*; program(s) used to solve structure: *SHELXS97* (Sheldrick, 2008 ▶); program(s) used to refine structure: *SHELXL97* (Sheldrick, 2008 ▶); molecular graphics: *SHELXTL* (Sheldrick, 2008 ▶); software used to prepare material for publication: *SHELXTL*.

## Supplementary Material

Crystal structure: contains datablock(s) I, global. DOI: 10.1107/S1600536812005703/is5027sup1.cif

Structure factors: contains datablock(s) I. DOI: 10.1107/S1600536812005703/is5027Isup2.hkl

Supplementary material file. DOI: 10.1107/S1600536812005703/is5027Isup3.cml

Additional supplementary materials:  crystallographic information; 3D view; checkCIF report

## Figures and Tables

**Fig. 1.**
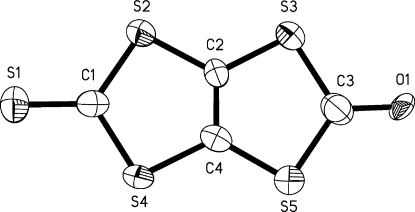
Molecular structure of the title compound. Displacement ellipsoids are drawn at 50% probability level.

**Fig. 2.**
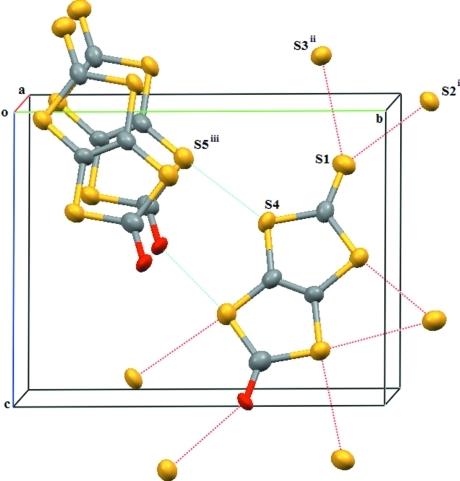
The packing pattern of the title molecules in crystal viewed down the a- axis, showing S···S and other intermolecular short contacts. [Symmetry codes: (i) 1/2 + x,-y + 2,-1/2 + z; (ii) x, y, -1 + z; (iii) 1/2 + x 1 - y -1/2 + z].

## supplementary crystallographic information

### Comment

Sulfur-rich compounds are well known as electronic donors in the field of organic molecular conductors, while their non-linear optical (NLO) properties are seldom reported. The synthesis of the title sulfur-rich compounds was reported by Schumaker and Engler (1977). Recently, we have re-synthesized the title compound by a new synthetic route and determined its X-ray structure.

The molecule adopts a planar conformation. All the C—S bond lengths are shorter than the length of C(*sp*^3^)—S single bond, showing the π-conjugated nature of the molecule. The molecules are parallel packed into columns along the *a* axis with a uniform spacing being 3.57 (1) Å. In addition to the above longitudinal π–π interactions, there are plenty of transverse short intermolecular S···S contacts. For example, S1···S2 [1/2 + *x*, 2 - *y*, -1/2 + *z*], S1···S3 [*x*, *y*, -1 + *z*], and S4···S5 [1/2 + *x*, 1 - *y*, -1/2 + *z*] distances are 3.314 (3), 3.482 (2) and 3.501 (2) Å, respectively.

The polar molecules crystallized into polar *Pn* space group. The angle [39.3 (1)°] between the two molecular dipole moments in the unit cell is not large, meaning that the crystal is well auto-polarized. The projection of both the molecular moments on the (010) plane is along the [1 0 -1.41] direction, which is also the direction of the macro polarization vector.

By the way, the molecular dipole moment has been calculated to be 0.8862 Bebye by using the Gaussian-03 programs (Frisch *et al.*, 2003), by the DFT method at the B3LYP/6–311(*d*) level. Meantime, the theoretical optimization for the molecular conformation by using the same Gaussian-03 programs indicates that the "free" molecule indeed adopts a perfect planar conformation with a strict C_2v_ symmetry.

The highly polar structure prompted us to carry out a frequency doubling experiment. When irradiated by the 1053 nm of laser pulses, the powder sample of the title crystal can emit 526.5 nm of green light, which is as strong as that of the urea powder crystals (as reference) and therefore has a remarkable 2-nd NLO effect.

### Experimental

We firstly synthesized bis(tetrabutylammonium)bis(1,3-dithiole-2-thione-4,5-dithiol) zincate precursor by the method reported by Wang *et al.* (1998). For the synthesis of the title compound, the above zincate precursor (2.3 g, 2.5 mmol) was dissolved in THF (50 ml). And then the triphosgen (1.40 g, 5.0 mmol) at 195 K was added in presence of N_2_. The solution was stirred overnight. An orange precipitate was obtained and dried *in vacuo*. And the final compound was purified by column chromatography using carbon disulfide as eluent, giving 0.46 g (86.7% yield) orange crystalline product. MS (EI): m/*z* 224.

### Refinement

There is no hydrogen atom in this structure. The reciprocal space showed a non-merohedral twin for the crystal used. All diffraction spots, except those of (0*kl*) kind, appeared in pairs. The diffraction spots on the (0*kl*) layer were supposed to be overlapped in pairs. By using the editing tools in RLATT, two reciprocal lattices corresponding to the two domains have been successfully separated and two P4P files been produced separately. After merging two P4P files into a new P4P file, data integration and absorption correction have been carried out. All the programs used are in the *APEX2* Software Suite (Bruker, 2005). The domain scale factor has been refined to be 0.293 for the second domain.

### Crystal data

**Table:**

C_4_OS_5_	*F*(000) = 224
*M**_r_* = 224.34	*D*_x_ = 1.997 Mg m^−^^3^
Monoclinic, *P**n*	Mo *K*α radiation, λ = 0.71073 Å
Hall symbol: P -2yac	Cell parameters from 1431 reflections
*a* = 3.9638 (2) Å	θ = 3.0–26.0°
*b* = 11.0211 (8) Å	µ = 1.47 mm^−^^1^
*c* = 8.7110 (7) Å	*T* = 294 K
β = 101.344 (5)°	Bar, orange
*V* = 373.11 (4) Å^3^	0.27 × 0.07 × 0.05 mm
*Z* = 2	

### Data collection

**Table:**

Bruker APEXII CCD diffractometer	1683 independent reflections
Radiation source: fine-focus sealed tube	1487 reflections with *I* > 2σ(*I*)
Graphite monochromator	*R*_int_ = 0.039
Detector resolution: 8.3 pixels mm^-1^	θ_max_ = 27.5°, θ_min_ = 1.9°
ω scans	*h* = −5→5
Absorption correction: multi-scan (*TWINABS*; Bruker, 2005)	*k* = −14→14
*T*_min_ = 0.697, *T*_max_ = 0.928	*l* = −11→11
8524 measured reflections	

### Refinement

**Table:**

Refinement on *F*^2^	Primary atom site location: structure-invariant direct methods
Least-squares matrix: full	Secondary atom site location: difference Fourier map
*R*[*F*^2^ > 2σ(*F*^2^)] = 0.044	*w* = 1/[σ^2^(*F*_o_^2^) + (0.0831*P*)^2^ + 0.0664*P*] where *P* = (*F*_o_^2^ + 2*F*_c_^2^)/3
*wR*(*F*^2^) = 0.130	(Δ/σ)_max_ < 0.001
*S* = 1.12	Δρ_max_ = 0.41 e Å^−^^3^
1683 reflections	Δρ_min_ = −0.45 e Å^−^^3^
92 parameters	Absolute structure: Flack (1983), 815 Friedel pairs
2 restraints	Flack parameter: 0.1 (3)

### Special details

**Table:**

Experimental. The twin operator is an 180° rotation about the c* axis and the twin law can be recognized as -1 0 0, 0 - 1 0, 0.873 0 1. The frequency doubling experiment indicates that it is impossible for the crystal to adopt the centrosymmetric *P*2_1_/*c* space group.
Geometry. All e.s.d.'s (except the e.s.d. in the dihedral angle between two l.s. planes) are estimated using the full covariance matrix. The cell e.s.d.'s are taken into account individually in the estimation of e.s.d.'s in distances, angles and torsion angles; correlations between e.s.d.'s in cell parameters are only used when they are defined by crystal symmetry. An approximate (isotropic) treatment of cell e.s.d.'s is used for estimating e.s.d.'s involving l.s. planes.
Refinement. Refinement of *F*^2^ against ALL reflections. The weighted *R*-factor *wR* and goodness of fit *S* are based on *F*^2^, conventional *R*-factors *R* are based on *F*, with *F* set to zero for negative *F*^2^. The threshold expression of *F*^2^ > σ(*F*^2^) is used only for calculating *R*-factors(gt) *etc*. and is not relevant to the choice of reflections for refinement. *R*-factors based on *F*^2^ are statistically about twice as large as those based on *F*, and *R*- factors based on ALL data will be even larger.

### Fractional atomic coordinates and isotropic or equivalent isotropic displacement parameters (Å^2^)

**Table:**

	*x*	*y*	*z*	*U*_iso_*/*U*_eq_	
S1	0.5018 (5)	0.86748 (19)	0.2126 (2)	0.0551 (5)	
S2	0.3162 (4)	0.91619 (15)	0.51687 (18)	0.0415 (4)	
S3	0.1270 (5)	0.82054 (14)	0.8209 (2)	0.0451 (4)	
S4	0.3021 (4)	0.66835 (13)	0.39376 (17)	0.0427 (4)	
S5	0.1178 (5)	0.56886 (16)	0.6947 (2)	0.0471 (4)	
O1	−0.0459 (13)	0.6265 (3)	0.9824 (4)	0.0394 (10)	
C1	0.3780 (16)	0.8219 (6)	0.3637 (8)	0.0373 (14)	
C2	0.216 (2)	0.8000 (6)	0.6361 (8)	0.0372 (13)	
C3	0.055 (2)	0.6625 (7)	0.8518 (8)	0.0487 (18)	
C4	0.211 (2)	0.6863 (6)	0.5810 (7)	0.0386 (14)	

### Atomic displacement parameters (Å^2^)

**Table:**

	*U*^11^	*U*^22^	*U*^33^	*U*^12^	*U*^13^	*U*^23^
S1	0.0732 (12)	0.0538 (10)	0.0417 (8)	−0.0058 (10)	0.0194 (8)	0.0051 (9)
S2	0.0544 (9)	0.0344 (7)	0.0386 (7)	−0.0034 (7)	0.0161 (7)	−0.0067 (6)
S3	0.0617 (10)	0.0400 (9)	0.0371 (7)	0.0003 (8)	0.0186 (8)	−0.0060 (7)
S4	0.0604 (10)	0.0291 (7)	0.0427 (8)	−0.0019 (7)	0.0205 (8)	−0.0101 (7)
S5	0.0614 (11)	0.0367 (8)	0.0478 (8)	−0.0023 (8)	0.0222 (8)	−0.0025 (7)
O1	0.066 (3)	0.0233 (18)	0.031 (2)	−0.004 (2)	0.015 (2)	0.0100 (16)
C1	0.034 (3)	0.032 (3)	0.046 (3)	0.000 (2)	0.009 (3)	−0.005 (2)
C2	0.046 (3)	0.038 (3)	0.032 (3)	−0.003 (3)	0.017 (3)	−0.002 (2)
C3	0.055 (4)	0.050 (4)	0.043 (4)	0.005 (3)	0.015 (3)	−0.006 (3)
C4	0.043 (3)	0.037 (3)	0.036 (3)	0.001 (3)	0.007 (3)	−0.009 (2)

### Geometric parameters (Å, º)

**Table:**

S1—C1	1.575 (7)	S4—C4	1.750 (6)
S2—C1	1.746 (6)	S5—C4	1.713 (7)
S2—C2	1.743 (6)	S5—C3	1.770 (7)
S3—C2	1.730 (7)	O1—C3	1.338 (8)
S3—C3	1.794 (8)	C2—C4	1.340 (7)
S4—C1	1.747 (7)		
			
C1—S2—C2	95.8 (3)	C4—C2—S2	117.6 (4)
C2—S3—C3	94.6 (3)	S3—C2—S2	124.8 (4)
C1—S4—C4	95.9 (3)	O1—C3—S5	126.6 (5)
C4—S5—C3	94.9 (3)	O1—C3—S3	119.9 (5)
S1—C1—S2	124.2 (4)	S5—C3—S3	113.5 (4)
S1—C1—S4	121.7 (4)	C2—C4—S5	119.3 (4)
S2—C1—S4	114.1 (4)	C2—C4—S4	116.6 (4)
C4—C2—S3	117.6 (4)	S5—C4—S4	124.1 (4)
